# Simulation-based low-dose, high-frequency plus mobile mentoring versus traditional group-based training approaches on *day of birth care* among maternal and newborn healthcare providers in Ebonyi and Kogi States, Nigeria; a randomized controlled trial

**DOI:** 10.1186/s12913-018-3405-2

**Published:** 2018-08-13

**Authors:** Emmanuel Ugwa, Emmanuel Otolorin, Mark Kabue, Gbenga Ishola, Cherrie Evans, Adetiloye Oniyire, Gladys Olisaekee, Boniface Onwe, Amnesty E LeFevre, Julia Bluestone, Bright Orji, Gayane Yenokyan, Ugo Okoli

**Affiliations:** 1 Jhpiego-an affiliate of Johns Hopkins University, 971 Reuben Okoya Crescent, Wuye District, Abuja, Nigeria; 2 Jhpiego-an affiliate of Johns Hopkins University, 1615 Thames St # 200, Baltimore, MD 21231 USA; 3 Department of Public Health, State Ministry of Health, Centenary City, Abakiliki, Ebonyi State Nigeria; 4 Department of International Health, Johns Hopkins Bloomberg School of Public Health, 615 N. Wolfe Street, Baltimore, MD 21205 USA; 5 The Johns Hopkins Biostatistics Center, Johns Hopkins Bloomberg School of Public Health, 615 N. Wolfe Street, Baltimore, MD 21205 USA; 6 School of Public Health and Family Medicine, Division of Epidemiology and Biostatistics, University of Cape Town, Western Cape, South Africa

**Keywords:** Low-dose, High-frequency, Traditional, Training, Simulation, M-mentoring, Health workers, Maternal, Newborn health, Nigeria

## Abstract

**Background:**

There is limited information from low and middle-income countries on learning outcomes, provider satisfaction and cost-effectiveness on the *day of birth care* among maternal and newborn health workers trained using onsite simulation-based low-dose high frequency (LDHF) plus mentoring approach compared to the commonly employed offsite traditional group-based training (TRAD). The LDHF approach uses in-service learning updates to deliver information based on local needs during short, structured, onsite, interactive learning activities that involve the entire team and are spaced over time to optimize learning. The aim of this study will be to compare the effectiveness and cost of LDHF versus TRAD approaches in improving knowledge and skill in maternal and newborn care and to determine trainees’ satisfaction with the approaches in Ebonyi and Kogi states, Nigeria.

**Methods:**

This will be a prospective cluster randomized control trial. Sixty health facilities will be randomly assigned for *day of birth care* health providers training through either LDHF plus mobile mentoring (intervention arm) or TRAD (control arm). There will be 150 trainees in each arm. Multiple choices questionnaires (MCQs), objective structured clinical examinations (OSCEs), cost and satisfaction surveys will be administered before and after the trainings. Quantitative data collection will be done at months 0 (baseline), 3 and 12. Qualitative data will also be collected at 12-month from the LDHF arm only. Descriptive and inferential statistics will be used as appropriate. Composite scores will be computed for selected variables to determine areas where service providers have good skills as against areas where their skills are poor and to compare skills and knowledge outcomes between the two groups at 0.05 level of statistical significance.

**Discussion:**

There is some evidence that LDHF, simulation and practice-based training approach plus mobile mentoring results in improved skills and health outcomes and is cost-effective. By comparing intervention and control arms the authors hope to replicate similar results, evaluate the approach in Nigeria and provide evidence to Ministry of Health on how and which training approach, frequency and setting will result in the greatest return on investment.

**Trial registration:**

The trial was retrospectively registered on 24th August, 2017 at ClinicalTrials.Gov: NCT03269240.

**Electronic supplementary material:**

The online version of this article (10.1186/s12913-018-3405-2) contains supplementary material, which is available to authorized users.

## Background

The competency of frontline birth attendants, particularly on the *day of birth care*, cannot be over-emphasized, as absence of knowledgeable and skilled service providers may cost lives or result in lifelong morbidity and its consequent socio-economic impacts. Research evidence has shown poor availability of skilled birth attendants in Nigeria and the need for evidence-based in-service training of all skilled birth attendants in order to improve competencies and maternal/newborn outcomes [1, 2].

Currently, in-service training of healthcare providers in Nigeria has assumed a traditional lecture-based, off-site or classroom approach of just a few service providers per site at a time. This approach to training can be costly, both in terms of individual provider costs as well as with regard to the opportunity costs of removing providers from clinical practice for extended period of time. Additional limitations in numbers of providers that can be trained in a single deployment can mean that only a small proportion of those engaged in delivery of a particular service receive added capacity building and ultimately, impacts on quality of facility services remain unchanged. When those providers return to their work stations, they often face challenges implementing new or updated skills because the entire team has not been reached which may constitute a barriers. Besides, providers’ turnover due to transfers, resignations, retirements and movement to other sectors also constitute barrier when they involve those who have been trained.

While it is important to increase coverage of maternal and newborn care, there is a need to improve the quality of services provided at health facilities. Evidence from a systematic review has shown that techniques such as simulation, practice and feedback are more effective than lecture and reading, and repetitive rather than single interventions result in better learning outcomes [3]. It is also known that settings similar to the work environment improved skill acquisition and performance of healthcare workers. These findings have driven Jhpiego’s shift into simulation and practice-based, shorter but repeated, workplace-based training. However, one of the limitations of this review was the severe lack of quality data from low- and middle-income countries (LMICs). Testing these approaches in LMICs is important to validate if these findings on technique, frequency, setting and media used to deliver instruction are replicable and feasible.

The LDHF approach uses in-service learning updates to deliver information based on local needs during short, structured, onsite, interactive learning activities that involve the entire team and are spaced over time to optimize learning. It also involves brief, ongoing activities (e.g., skills practice, team drills, games, and quality improvement activities) at the job site to sustain learning and support clinical decision-making.

The principles of the LDHF approach include:

1. Competency-focused learning activities concentrates on what providers “need to know”—eliminating what is “nice to know.”
2. Simulation- and case-based learning focuses on skills practice, problem-solving, role-play, and other interactive exercises. Dosing and frequency depend on topic, extent of the learning gap, and learner characteristics.
3. Appropriately spaced, brief periods of learning deliver targeted information in 1 day or over several days.
4. Team-focused training ensures that all providers have updated clinical practice and can work together to implement improvements in care.
5. Facility-based training decreases absenteeism, improves teamwork, addresses onsite barriers, and promotes changes to provider performance.
6. Ongoing practice and quality improvement activities reinforce learning and transfer to clinical practice.
7. Facility-based peer staff coach others as they practice or engage in interactive exercises after learning to increase compliance and improve performance and outcomes.

Gaps exist in high-quality evidence from Nigeria and other developing countries on the effectiveness of simulation-based LDHF/m-Mentoring learning approaches in improving maternal and newborn health and trainees’ satisfaction. Even in developed countries where these approaches have been tested, the studies are limited by small sample sizes. Therefore it is important that future research on health worker capacity building in Nigeria and other LMICs should include the testing of techniques to enhance knowledge, skills and satisfaction among healthcare workers using appropriately designed and statistically powered studies.

Factors known to affect skills and knowledge acquisition including motivation and learning environment [4], team leadership and communication [5], and use of simulators to enhance skills retention for low-frequency procedures [6] and others have been explored by various researchers. It has been shown that a strong health workforce leadership is an important factor to ensure motivation and create an enabling learning environment [7–9].

Apart from high cost of training generally [10], traditional off-site training can lead to service disruptions due to absenteeism to attend trainings and loss of personal income [11]. Apparently therefore, different training strategies will be associated with different costs and there is need to combine cost effectiveness, learning outcomes, and alignment with national strategy in order to inform better policy decisions [12, 13].

Maternal and Child Survival Program (MCSP), a United States Agency for International Development (USAID) multi-country cooperative agreement serves as conduit for bringing innovations into healthcare practice in Ebonyi and Kogi States, in South-East and North-Central Nigeria respectively with special emphasis on the *‘Day of Birth Care’*. The core package includes evidence-based interventions such as use of the partograph, administration of uterotonic, essential newborn care, newborn resuscitation, and the use of magnesium sulphate for severe pre-eclampsia and eclampsia. In addition to supporting clinical innovations, MCSP has an implementation research agenda for process innovations to test the effectiveness of different approaches to improve maternal and newborn health care delivery services. For example, through control and intervention demonstration learning groups, MCSP endeavors to build evidence to support the shift from group based training outside of the health facility to LDHF approach.

The authors hypothesized that simulation-based LDHF/m-Mentoring will result in better satisfaction, knowledge and skill outcomes as well as will be more cost effective compared to a group-based approach among birth attendants in Kogi and Ebonyi states.

## Methods/Design

### Study aim

The aim of this study will be to compare the effectiveness and cost of onsite LDHF plus m-Mentoring training approach versus the traditional offsite group-based training approach in improving knowledge and skill in maternal and newborn *day of birth* care and to determine trainees’ satisfaction with the approaches in Ebonyi and Kogi states of Nigeria.

The specific objectives will be to:

1. Compare knowledge and skills learning outcomes between the two groups of birth attendants trained through the simulation-based LDHF/m-Mentoring versus group-based training approaches in Kogi and Ebonyi states over 12-months.
2. Assess the trainees’ levels of satisfaction with a simulation-based LDHF/m-Mentoring and group-based training in the selected facilities in Kogi and Ebonyi state over 12-months.
3. Determine the cost and cost-effectiveness of LDHF/m-Mentoring and group-based training approaches in improving skills of birth attendants in the selected facilities in Kogi and Ebonyi state over 12-months.

### Study design

The study will be a prospective cluster randomized control trial. It will include healthcare providers in 60 health facilities in Kogi and Ebonyi State randomly assigned to either LDHF (intervention) or TRAD (control) arms. For both study arms, skills and knowledge assessments will be done by independent assessors who will be blinded to the training approaches used. This will be at three time points post-training (immediate post training day, at 3 months 12 months) using MCQs (see Additional file 1) and OSCEs. Cost and trainees’ satisfaction level with training approach is determined using satisfaction (quantitative) survey. Qualitative data will also be collected through focus group discussions (FGD) and in-depth interviews (IDIs) at 12 months for the LDHF arm.

### Study setting

**Fig. 1:**
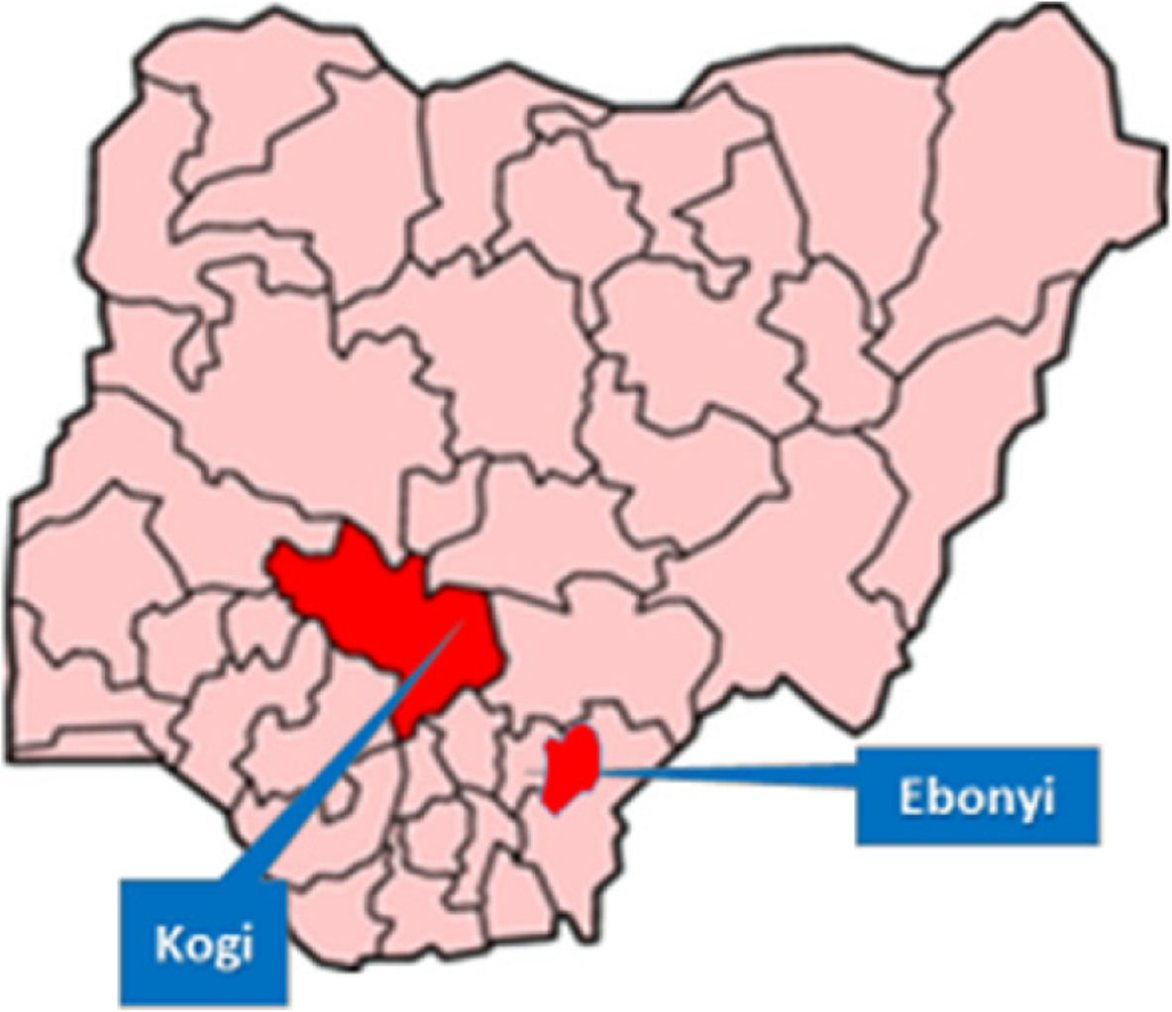
Geographical locations of project States

MCSP is working in selected health facilities in Ebonyi and Kogi States in Nigeria to improve the quality of care received by mothers and newborns on the day of birth using high-impact evidence-based lifesaving interventions. Nigeria with a population of 185.7million has 6 geopolitical zones of which Ebonyi with a population of 2.79 million is in the South-east and Kogi with a population of 4.3million is located in the Northcentral zone (please see Fig. 1). At the time of the study, MCSP was supporting 120 health facilities, of which 60 will be selected to be part of the study.

### Eligibility criteria

#### Health facilities

Selection of health facilities will be from the 120 supported by MCSP in the two states in all three senatorial geopolitical zones per State and will include all three levels of healthcare delivery namely primary (primary health care/private clinics), secondary (general/mission) and tertiary.

#### Service providers

Individual study participants will be drawn from those providing maternal and newborn health in the selected health facilities in the two states. Participating health workers will include doctors, nurses, midwives and community health extension workers. The service providers should have spent at least 6 months in maternal and/or newborn care services, and should be available to participate in the training from the beginning to the end (12-month period).

### Randomization

The unit of randomization will be the health facility. All 60 facilities will be divided into 9 strata by 3 geopolitical zones and by level of healthcare delivery; each stratum is randomized to either control or intervention using randomly permuted blocks in ratio of 1:1 to achieve balance in type of facility and location by treatment arm. Health facilities will be matched prior to randomization and will be grouped as either receiving simulation-based LDHF/m-Mentoring or traditional training. As skilled birth attendants are few in the health facilities, all health providers working in maternity or newborn units who meet the inclusion criteria will be selected from the randomized health care facilities. Arm 1 will include birth attendants who will be trained using LDHF approach while arm 2 will be those who will be trained using TRAD approach.

### Sample size

We will calculate the average number of providers who will be included from each of the 60 facilities (or 30 facilities in each arm) using a test of two proportions for a binary indicator of a present competency score. Competency will be assumed at a score of 80% or higher score on both the knowledge and skills assessments. We assumed that the proportion of competent providers will be 50% in the control group. The goal is to have at least 80% power to reject a null hypothesis that the proportions are equal across the two arms against the alternative hypothesis of a minimum effect size of 20 percentage point difference in proportions of competent providers. The test of hypothesis will be performed at 0.05 level of significance. Sample size was calculated using PASS statistical software [14]. Since some facility-level factors that are shared by the providers working in the same facility can influence the competency level, we will assume 0.05 within facility correlation of competency levels across providers in a given health facility**.** Assuming that an average of four (04) service providers sampled per health facility, a total of 240 providers across the two groups will be needed so that there will be at least 80% power to detect a 20 percentage point difference in competence level between the two arms (assessment at 3 months) at 0.05 level of statistical significance. The final sample size will be 300 or 150 providers per study arm to account for potential 20% drop-out at the time of post-training assessment. The participants for each facility will include the maternity unit head wherever possible and two others; to ensure the other team members receive the necessary support they need to practice the competencies. Study participants will be administered a single survey comprised of modules on characteristics, knowledge, and costs incurred in addition to a competency assessment prior to training and at three time points following training (end of training day, 3, 12 months post-training).

### Training approach 1 and data collection: Simulation-based LDHF/m-mentoring training of participants - group 1 or intervention group

The training for the LDHF arm will be for the entire team of service providers available at the health facility, but only those who will meet the study inclusion criteria will be assessed. After oral consent is obtained, the participants will take pre-training assessments consisting of MCQs and OSCEs through use of manikins, to assess their baseline knowledge and skills. The MCQ contains a set of multiple choice questions to test trainees’ knowledge. OSCE involves the use of validated checklists to evaluate trainees’ demonstration of clinical skills to ensure that each step is correctly and completely carried out. The assessments will test their knowledge and skills on conduct of normal delivery, active management of the third stage of labor (AMTSL), neonatal resuscitation, case management of pre-eclampsia and eclampsia (PEE) and management of PPH (e.g. manual removal of placenta, internal bimanual uterine compression and compression of the abdominal aorta). The training will be divided into two “low-dose” training courses of 4 days each, with additional time for assessment as needed and will be conducted at the health facilities using the adapted basic emergency obstetrics and newborn care (BEmONC) package repeated after 1 month. The training techniques will be modified to shift the emphasis to practice. The time spent on lectures will be reduced and time spent on hands-on practice will be increased. The Peer Practice Coordinators (PPCs) will receive technical update in LDHF including the use of session plans, case scenario and MamaNatalie/NeoNatalie models to conduct simulation practices. At the end of the four-day training, the participants will undergo an immediate post-training assessment which includes MCQs and OSCEs. The questions answered correctly and procedure done competently is scored over a total of 100%. A test score of ≥80% is accepted as level of competence. The pre-training and immediate post-training assessments results will be compared. During the one-month intervals between training courses, health care providers will have the opportunity to practice what they learned and reinforce their competencies through high-frequency simulation-based practices of 2–3 times weekly. The PPCs will complete the practice log. In addition to the simulation exercises, all the trained providers in the LDHF arm will participate in mobile Mentoring (mMentoring), which consists of receiving weekly reminder messages and quiz questions on the topics reviewed via SMS messaging. Also the PPCs will receive structured, monthly half-hour mentoring calls from a trainer/master mentor that provide remote support, answering questions, providing guidance and reinforcing key messages. Skills and knowledge assessments will be done at three time points post-training (immediate post training day, at 3 months 12 months). Trainees’ satisfaction with the simulation-based LDHF/m-Mentoring training approach will also be determined using satisfaction (quantitative) survey.

Qualitative data will be collected through six (6) focus group discussions (FGD) comprising 8–10 similar participants per group purposively selected from among the trainees at 12 months based on their availability and experience. The FGD focuses on the experiences and satisfaction of trainees with LDHF training approach, and high-frequency practice sessions with simulators, mobile mentoring sms and quizzes, opinions about changes in clinical practice and outcomes and overall impressions of the LDHF approach and what could be improved. The FGD will last 60–90 min. In-depth interviews (IDIs) will also be conducted with PPCs and trainers at 12 months post-training by study staff. The IDIs for PPCs will aim at hearing their insights and experience managing simulator practice sessions for their facility, interacting and working with the trainers/master mentors, m-Mentoring, changes in clinical practice and outcomes, success and challenges and overall impression about LDHF approach. For the trainers, their thoughts and experience with the LDHF training approach, successes and challenges with mobile mentoring and supporting peer practice coordinators, opinions on the effectiveness of m-Mentoring, confidence in their role as trainers, successes and challenges in collecting data will be sought. Each IDI will last about 45 min and is audio recorded.

### Training approach 2 and data collection: Traditional group training of participants – Group 2 or control group

The traditional training approach will consist of 8 days of lectures with practice sessions on simulators, off-site the participants’ workplace, usually in a hotel. Oral consent will be obtained, the participants will take pre-training assessments consisting of MCQs and OSCEs through use of manikins, to assess their baseline knowledge and skills respectively. The assessments will test their knowledge and skills on conduct of normal delivery, AMTSL, neonatal resuscitation, case management of PEE and management of PPH (e.g. manual removal of placenta, internal bimanual uterine compression and compression of the abdominal aorta). This group then receives lectures and practice sessions on conduct of normal delivery, AMTSL, neonatal resuscitation, case management of PEE and management of PPH. The Nigerian BEMONC package and clinical observation checklists are used during the training sessions. The group-based training approach will include training sessions consisting of 8 days of lectures and fewer practice sessions using manikins (MamaNatalie/NeoNatalie) offsite and not at the trainees’ place of work. Table 1 outlines the basic similarities and difference between the two groups. At the end of the eight-day training, the participants will undergo an immediate post-training assessment which includes MCQs and OSCEs. As described among arm 1, the questions answered correctly and procedure done competently will be scored out of a total of 100%, assessments results will be compared and trainees satisfaction survey will be conducted.

**Table 1:** Comparison of the Simulation-based LDHF and Group-based study arms

Parameters of the interventions received	Simulation-based LDHF Arm	Group-based Arm
Training covers BEMONC	Yes	Yes
Training is onsite	Yes	No
More frequent practise sessions using manikins during training	Yes	No
Post-training Practise Sessions with manikin in facility	Yes	No
Presence of PPCs	Yes	No
Use of mobile phone for post-training support	Yes	No
All courses divided into two phases which are 1 month apart	Yes	No

### Cost effectiveness analysis

As part of LDHF and TRAD evaluation activities, we will prospectively monitor the costs of both approaches across two perspectives: (1) incremental program costs, and (2) costs to providers/ health system. Incremental program costs include costs incurred by Jhpiego in the implementation of LDHF and/or TRAD training across three distinct phases: development, start-up and ongoing implementation support. Costs will be collected according to an ingredients approach and differentiated according to capital (costs with a life expectancy of greater than 1 year) and recurrent costs. The former will be annualized drawing from standardized WHO-CHOICE life expectancy estimates and discounted at 3%. Costs incurred by providers / health system primarily focus on the opportunity costs of in-service training to providers and the health system. These will be captured through questions imbedded into knowledge surveys. Efforts to determine the value for money of LDHF vs. TRAD training will draw from data on the incremental costs and effects of program activities. Incremental costs will be collected throughout the 1-year analytic time horizon of the program and presented in 2018 United States Dollars. Incremental effects will be drawn from data mentioned above and utilize composite scores of knowledge and competency.

### Data analysis

#### Primary outcome

Clinical competency level in BEmONC skills of service providers at 3 months post-training.

#### Secondary outcomes

- Retention in clinical competency level in BEmONC skills as assessed through OSCEs and MCQ scores at 12 months post-training.
- Service provider satisfaction with a simulation-based LDHF/m-Mentoring and group-based training approaches.
- Direct cost and cost-effectiveness of LDHF/m-Mentoring and group-based training approaches.

#### Descriptive analysis

Absolute numbers and simple percentages will be used to describe categorical variables. Measures of central tendency (mean and median) as well as dispersion (range and standard deviation) will also be determined. The unit of analysis will be the service providers. First, we will check the balance between the two treatment arms in terms of potential confounding factors, such as provider- (age, experience) or facility-(location, level of healthcare delivery, ownership)-level characteristics. In addition, pre-test scores is compared between the groups, overall and stratified by location and level of healthcare delivery. Percentage of those achieving the required competency level (≥80% post-training scores in each of MCQ and OSCE) for both groups 1 and 2 is calculated. This will be done for pre-training and subsequent post-training follow-up assessments. Composite scores will be computed for selected variables to determine areas where service providers have good skills as against areas where their skills are poor.

#### Inferential analysis

Generalized linear model with robust facility-level variance will be used to test the differences between arms in MCQ scores, OSCE scores and satisfaction outcomes at 3 months using a group indicator as the main predictor in the model. Additionally, we will adjust for provider- and facility-level characteristics that show imbalance between arms at baseline and can be strongly correlated with the outcome scores. A *p*-value < 0.05 will be considered statistically significant. The number of short messages sent is assessed for trend of the reply and correct answers. The frequency and trend of mentorship call will also be assessed. The model described for the primary outcome is used to look at the outcomes at other time points. In addition, we will develop a longitudinal model that assess the change in scores (or competency level) over time post-training. This model is appropriate, since it accounts for correlation within a provider over time as well as within-facility correlation of scores. Generalized linear population-average model estimated using generalized estimating equations (GEE) with working exchangeable correlation structure is used. The amount of missing data is assessed for each variable and overall for the sample. If more than 5% of data are missing, multiple imputation procedure is used assuming missing at random. Composite scores is computed for the satisfaction survey. A Decision Tree will be used to compare incremental costs and incremental effects by provider across the two study arms. One-way, multi-way and probabilistic sensitivity analyses are conducted to assess uncertainty.

Qualitative data analysis: Notes and transcripts will be entered into Atlas-ti version 8 software. Content analysis of the discussions will be undertaken to generate themes of interest along which the analysis will be done. Analysis will be by themes across location and classifications of the groups.

## Discussion

Findings from a similar study done in Ghana by Jhpiego/Ghana Health Services, using a low-dose, high-frequency, simulation and practice-based training approach, including repeated practice, mobile mentoring and the integration of simple quality assurance approaches have early promising results including improved skills, health outcomes and cost effectiveness [10]. Using a cluster randomized controlled trial design the authors hope to replicate similar result by evaluating the approach in Nigeria and provide evidence to MOH on how and which training techniques, frequency and setting will result in the greatest return on their investment. The current study also goes further to determine the cost effectiveness of and trainees’ satisfaction with the two models. Considering that resources are limited especially in developing countries where there is a limited health budget, the prospect for improved productive efficiency of the health workforce should also be weighed against training cost. Therefore comparative cost of developing and implementing any training methodology needs to be closely studied in order to justify the costs if it results in better learning and possibly health outcomes. The project’s goal will be to translate Nigerian evidence into routine practice and innovations that work in the Nigerian context will be scaled up through the adoption of national policies and implementation plan. This implementation research agenda for process innovations is to test the effectiveness of different approaches to improve maternal and newborn health care delivery services. Through control and intervention demonstration learning groups, MCSP will work to build evidence to support the shift from group based training outside of the health facility to LDHF approach demonstrated.

## Additional file

Additional file 1: Multiple choice questions testing basic knowledge of maternal and newborn health. (DOCX 34 kb)

## Abbreviations

AMTSL: Active management of third stage of labor
BEmONC: Basic Emergency Obstetrics and Newborn Care
IRB: Institutional Review Board
LDHF: Low-dose, high-frequency
MCQ: Multiple choice questions
MCSP: Maternal and Child Survival Program
m-Mentoring: Mobile mentoring
MNCH: Maternal Newborn and Child Health
NHREC: Nigerian Health Research and Ethics Committee
OSCE: Objective structured clinical examination
PEE: Pre-eclampsia/Eclampsia
PPCs: Peer practice coordinators
PPH: Primary postpartum haemorrhage
SMS: Short Messages
