# Supplementary material for: Simulation-based low-dose, high-frequency plus mobile mentoring versus traditional group-based training approaches on day of birth care among maternal and newborn healthcare providers in Ebonyi and Kogi States, Nigeria; a randomized controlled trial
Source: BMC Health Serv Res. 2018 Aug 13;18:630. doi: 10.1186/s12913-018-3405-2 (PMC6090683; doi:10.1186/s12913-018-3405-2)
Supplement: Supplementary file 1 — Multiple choice questions testing basic knowledge of maternal and newborn health. (DOCX 34 kb) [file 12913_2018_3405_MOESM1_ESM.docx]

Date ___/___/____ (dd/mm/yyy)

**Tool A- Pre-Post Knowledge Assessment**

***Instructions: Please circle the letter of the correct response.***

## INFECTION PREVENTION

1. To decontaminate soiled items after delivery, soak them in 0.5% chlorine solution for
   1. 5mins
   2. **10mins**
   3. 15mins
2. Bacterial endospores which cause tetanus and gangrene are reliably killed by
   1. Savlon
   2. Boiling
   3. **Sterilization**
3. Hand washing is indicated before
   1. Examining clients
   2. Putting on sterile or high-level disinfected gloves
   3. **Both a and b**
4. To make 0.5% solution of chlorine from a concentrated solution of 10%, add one part of chlorine to how many parts of water

| a. | 10 |
| --- | --- |
| b. | 15 |
| **c.** | **19** |

1. When should you wash your hands?
   1. After exposure to blood and body fluids
   2. Before putting on gloves and after removing them
   3. **a and b**

**LABOR AND DELIVERY**

1. When fetal heart rate goes below 100bpm, the first action you should take is to position the mother
   1. On her back
   2. **On her left side**
   3. Squatting
2. How often should the fetal heart be checked in active labor and recorded on the modified WHO partograph?
   1. Every fifteen minutes
   2. **Every thirty minutes**
   3. Every sixty minutes
3. Cervical dilatation plotted to the right of the alert line indicates
   1. Satisfactory progress of labor
   2. **Unsatisfactory progress of labor**
   3. The beginning of the active phase
4. When a woman is admitted during the active phase of labor, cervical dilatation is initially plotted on the partograph
   1. To the left of the alert line
   2. To the right of the alert line
   3. **On the alert line**
5. Before applying controlled cord traction (CCT) during active management of labor (AMTSL)
   1. Apply pressure on the fundus
   2. **Check for a second baby and give oxytocin IM within one (1) minute of birth**.
   3. Wait for gush of blood and elongation of the cord
6. When should a skilled provider be present at a birth?
   1. If problems occur
   2. **At every birth**
   3. Whenever called by an attendant
7. Respectful maternity care includes
   1. Speaking to the woman and her family using medical terminology so she learns it
   2. Assuming she understands everything you are saying
   3. **Encouraging the woman to have a support person in labor**
8. The most important step in active management of third stage of labor is:
   1. **Administration of a uterotonic drug**
   2. Control cord traction to remove the placenta
   3. Massage of the uterus
9. The uterotonic drug of choice for active management of third stage of labor is:
   1. Ergometrine
   2. Misoprostol
   3. **Oxytocin**
10. The anticonvulsant drug of choice for treatment of eclampsia is:
    1. Diazepam
    2. **Magnesium Sulphate**
    3. Phenobarbitone
11. The commonest cause of postpartum hemorrhage is:
    1. Perineal laceration
    2. Retained placenta
    3. **Uterine atony**
12. Basic emergency obstetric and newborn care (BEmONC) includes the following signal functions EXCEPT:
    1. Assisted vaginal delivery (e.g. vacuum extraction)
    2. Administration of parenteral antibiotics
    3. **Blood transfusion**

## ESSENTIAL NEWBORN CARE AND NEWBORN RESUSCITATION

1. Which of the following is approved in Nigeria for application to the umbilical cord at birth, to help prevent cord infection?
   1. Nothing should be applied to the cord
   2. Methylated spirits
   3. **Chlorhexidine gel**
2. You have provided ventilation with Ambu bag and mask for 5 minutes. The baby’s chest is moving but the heart rate is about 70bpm. What should you do?
   1. Continue ventilation for another 10 minutes while observing
   2. **Continue ventilation, activate the emergency plan, and seek advice from a specialty facility.**
   3. Stop ventilation and observe to see if the heart rate improves.
3. The first step in thermal protection for the newborn includes
   1. **Drying the baby thoroughly immediately after birth**
   2. Drying the baby thoroughly after the cord has been cut
   3. Covering the baby with a clean, dry cloth immediately after birth
4. Immediate care for a normal newborn includes
   1. Skin-to-skin contact followed by placing the baby in a warming incubator
   2. **Drying the baby, removing the wet cloth, placing the baby skin-to-skin with the mother and covering the baby with a clean, dry cloth and hat**
   3. Stimulating the baby by slapping the soles of the baby’s feet
5. What will happen to the baby who is not dried, but placed on a cloth beside the mother?
   1. **The baby will become cold**
   2. The baby will stay warm
   3. The baby will get dry
6. What can happen when a baby inhales meconium?
   1. **The baby can have breathing problems**
   2. Meconium rarely causes breathing problems
   3. The baby will vomit it out
7. What should you do when a baby cries after birth and breathes quietly and regularly?
   1. Provide help to breathe
   2. **Give routine care**
   3. Refer the baby to the pediatrician
8. What should you do when a baby does not cry, breathe nor move at birth, and is limp?
   1. **Provide help to breathe**
   2. Do nothing for this baby
   3. Refer the baby to the pediatrician
9. What can you do to encourage breastfeeding?
   1. Put baby to breast only when baby cries
   2. Give warm tea and to both mother and baby
   3. **Keep mother and baby together and counsel on attachment to the breast**
10. How long should you wait to clamp or tie the umbilical cord of a crying baby?
    1. Clamp or tie the cord immediately
    2. **Wait 1 to 3 minutes (or until pulsations stop) to clamp or tie and cut the cord**
    3. Wait for 5 minutes to clamp or tie and cut the cord
11. What should you do if you notice bleeding from the umbilical cord even though a tie or clamp is in place?
    1. **Place another tie or clamp between the first one and the baby’s skin**
    2. Place another tie or clamp after the first one
    3. Apply pressure on the cord to stop the bleeding
12. If a baby is not breathing well after thorough drying and rubbing the back once or twice, you should
    1. Give more stimulation
    2. Keep rubbing the back
    3. **Ventilate with bag and mask**
13. Which baby is breathing well?
    1. **A baby who is breathing quietly and regularly**
    2. A baby who takes one deep breath followed by a long pause
    3. The baby who is gasping
14. How do you select the correct mask?
    1. **Select the mask that covers the chin, mouth and the nose but not the eyes**
    2. Select the mask that covers the chin, mouth, nose and eyes
    3. Select the mask that covers the mouth and the nose but not the eyes
15. Which cloth will keep the baby warmer during ventilation?
    1. A cloth soaked in warm water
    2. **A warm dry cloth**
    3. A cloth soaked in cold water
16. What helps you to move air into the baby’s lungs during ventilation?
    1. A fully extended position of the head
    2. A flexed position of the head
    3. **A good seal between the mask and the face**
17. How should you position the baby’s head to help open the baby’s airway?
    1. **Slightly extend the head**
    2. Hyperextend the head
    3. Keep the head straight
18. You are giving a baby ventilation with a bag and mask. The baby is gasping. What should you do?
    1. Stop ventilation and observe closely with mother
    2. **Continue ventilation**
    3. Observe to see if baby will eventually die
19. A baby begins to breathe well after 30 seconds of ventilation with bag and mask. How will you care for this baby?
    1. **Provide routine care and monitor the baby closely with the mother**
    2. Observe the baby in a cot
    3. Continue to ventilate for up to 1 minute
20. What should you do first when a baby does not breathe after ventilation?
    1. **Call for help**
    2. Check the umbilical cord for pulsation and continue ventilation
    3. Discontinue ventilation and call for help
21. A baby’s chest does not move with ventilation. What should you do first?
    1. Stimulate the baby
    2. Squeeze the bag harder to give a larger breath
    3. **Reapply the mask to the face and reposition the head**
22. You are alone and ventilating a baby with a bag and a mask. When should you stop to check the heart rate?
    1. After every 10 breaths with the ventilation bag
    2. **After 1 minute of ventilation**
    3. After 5 minutes of ventilation
23. What should you do next if you cannot feel the pulsations in the umbilical cord to count the heart rate?
    1. Continue to ventilate the baby
    2. **Listen to the heartbeat with a stethoscope**
    3. Do nothing more. The baby is dead
24. A baby has been ventilated for more than 3 minutes with bag and mask. The heart rate is 120 beats per minute. The baby is not breathing. What should you do next?
    1. **Slowly decrease the rate of ventilation and watch for breathing**
    2. Gradually increase the rate of ventilation and watch for breathing
    3. Stop the ventilation and wait for at least 1 minute to see if the baby breathes
25. A baby has been ventilated for 10 minutes with a bag and mask. The baby is now breathing and has a heart rate of more than 100 beats per minute. What care does this baby need?
    1. Routine care with mother
    2. **Close monitoring with specialty consultation or referral**
    3. Keep in a warmer
26. After 10 minutes of ventilation with a good chest movement, the baby is not breathing and there is no heart rate (no cord pulse, no heartbeat by stethoscope). What should you do?
    1. Stop ventilation for a while and continue again
    2. Continue ventilation for another 10 minutes
    3. **Stop ventilation. The baby is dead.**
27. A baby needed ventilation with bag and mask. S/he is breathing fast and cannot breastfeed. What should you do?
    1. Leave the mother and baby alone to rest
    2. **Explain to the mother and birth companion that the baby needs specialty care**
    3. Explain the baby’s condition to the birth companion
28. A premature baby is to be taken to the district hospital with breathing difficulty. How should you advise the mother?
    1. **Advise her to go with her baby if possible**
    2. Advise her to stay away from her baby
    3. Advise her not to travel for at least a week
